# Issues and complexities in safety culture assessment in healthcare

**DOI:** 10.3389/fpubh.2023.1217542

**Published:** 2023-06-15

**Authors:** Louise A. Ellis, Emma Falkland, Peter Hibbert, Siri Wiig, Eline Ree, Timothy J. Schultz, Christy Pirone, Jeffrey Braithwaite

**Affiliations:** ^1^Centre for Healthcare Resilience and Implementation Science, Australian Institute of Health Innovation, Faculty of Medicine, Health, and Human Sciences, Macquarie University, Macquarie Park, NSW, Australia; ^2^IIMPACT in Health, Allied Health and Human Performance, University of South Australia, Adelaide, SA, Australia; ^3^SHARE—Centre for Resilience in Healthcare, Department of Quality and Health Technology, Universitetet i Stavanger, Stavanger, Norway; ^4^Flinders Health and Medical Research Institute, Flinders University, Bedford Park, SA, Australia; ^5^Southern Adelaide Department of Health, Adelaide, SA, Australia

**Keywords:** safety culture, safety climate, patient safety, survey, healthcare benchmarking

## Abstract

The concept of safety culture in healthcare—a culture that enables staff and patients to be free from harm—is characterized by complexity, multifacetedness, and indefinability. Over the years, disparate and unclear definitions have resulted in a proliferation of measurement tools, with lack of consensus on how safety culture can be best measured and improved. A growing challenge is also achieving sufficient response rates, due to “survey fatigue,” with the need for survey optimisation never being more acute. In this paper, we discuss key challenges and complexities in safety culture assessment relating to definition, tools, dimensionality and response rates. The aim is to prompt critical reflection on these issues and point to possible solutions and areas for future research.

## 1. Background

Each year, millions of patients worldwide suffer injuries, disabilities, and even death due to unsafe medical practices (1–3). A recent retrospective cohort study from 11 US hospitals identified at least one adverse event in 24% of hospital admissions (1–3). This has led to the increasing recognition of the concept of safety culture, as it is argued to form the foundation for the safe delivery of high-quality healthcare (4).

The term “safety culture” was first conceptualized by the International Nuclear Safety Advisory Group as a response to the defective processes that contributed to the 1986 Chernobyl nuclear power plant disaster (5). Since then, the concept has been embraced by several high reliability, safety critical industries, such as aviation and nuclear power, and is considered a pivotal factor for the safety performance of organizations. More recently, the focus on building a culture of safety moved to the healthcare industry (6), where the promotion of a culture of safety has become one of the pillars of the patient safety movement (2). With growing recognition of the importance of safety culture in healthcare, the need for robust assessment measures became evident (7), and in turn, initiatives to improve and assess safety culture proliferated (8, 9). In part, growth in this area has developed in parallel with increasing external pressure from accreditation, regulation, and other safety agencies for healthcare organizations to undertake regular safety culture assessments (10).

Safety culture assessment is used in healthcare for several key reasons, chief of which are: (1) to diagnose safety culture to identify areas for improvement and raise awareness about patient safety; (2) to evaluate patient safety interventions and track change over time; (3) to conduct internal and external benchmarking; and (4) to fulfill directives or regulatory requirements (2). Notably, improving safety culture has become a significant priority for the Organization for Economic Co-operation and Development (OECD), especially as healthcare systems have faced additional safety concerns due to the implications of the COVID-19 pandemic (11). In 2020, the OECD compared the safety culture results of 16 countries, in an attempt to harmonize approaches, standardize methodologies, improve comparability of safety culture data over time, and to contribute to international benchmarking efforts (11). This work has revealed the heterogeneous nature of how healthcare staff perceive patient safety in their work environments, and has afforded opportunities to best practices regarding efforts to improve safety culture (11). Despite such efforts, several challenges persist in the measurement and intervention of safety culture that must be considered, including variability in definitions, tools, dimensionality, and response rates. In this paper, we have drawn on recent literature and experiences in patient safety culture assessment to critically appraise each of these issues and then suggest possible solutions and areas for future research.

## 2. Challenges

### 2.1. Definitional issues

Safety culture is arguably a poorly articulated concept, whereby many different definitions are apparent both within, and outside of, the healthcare domain (12). For example, there have been over 51 distinct definitions proposed, leading some researchers to refer to the concept as having, “the definitional precision of a cloud” (13, 14). This lack of cohesion has led to the development of various frameworks, each built upon varying definitions in how to conceptualize and extract meaning from the concept (14, 15).

Compounding the issue of definitional equivocality, many researchers also mark a distinction between safety *climate* and safety *culture*. While safety culture is argued to denote more longstanding, engrained behaviors, practices, beliefs and values within an organization, safety climate is proposed to embody people's *perceptions* of their organization (its procedures, practices, and the kind of behaviors that are tolerated or rewarded) at a given time (16–18). Following this, some argue that it is easier to measure safety climate than culture; if climate is considered a more temporal state of safety at a discrete point in time, it is thus more measurable. However, many others use the terms safety culture and safety climate interchangeably within the research literature (14, 19, 20). For the purposes of this paper, we use the term safety culture to include both culture and climate.

The most commonly used definition of safety culture was proposed by the Health Safety Commission (1993): “The product of individual and group values, attitudes, competencies and patterns of behavior that determine the commitment to, and the style and proficiency of, an organization's health and safety programmes” (p. 339) (12). However, some suggest that the broadness of such a definition weakens its scientific utility, indicating that much greater precision is required (21). So here lies another challenge; although the Health Safety Commission's definition may provide some guidance on which constructs to examine when assessing safety culture, the specific values, attitudes, competencies and behaviors and how to measure them is still not clear (15). Consequently, this has led to the development of many different tools, and in particular surveys, with each attempting to measure the complexities of safety culture (4, 12, 22). Indeed, surveys are particularly attractive as they are practical and time-efficient tools for gathering large amounts of data in a reliable and reproducible manner; thus supporting comparison and international benchmarking efforts. The anonymity usually involved in this form of data collection also makes them appealing for quality improvement, as they facilitate the contributions of staff who may be uncomfortable expressing their views openly (14, 15).

### 2.2. Variability in tools

Growing interest in safety culture has been accompanied by a proliferation of tools, each deriving from differing conceptualizations of safety culture (23). At least 220 different safety culture or safety climate surveys have been identified across industry sectors (24). The multitude of surveys has led to numerous systematic reviews of the available tools both within and outside of healthcare. Within healthcare, there is wide variability in the number of dimensions (ranging from one to 12) and items (ranging from 10 to 74 items) that the tools contain, and their validity, and adaptability for use in multiple settings (8), with no one tool emerging as the gold standard (12). The most widely employed surveys employed in safety culture research, and arguably the most validated, as identified in a recent safety culture review, are the Hospital Survey on Patient Safety Culture (HSOPS) (25), the Safety Attitudes Questionnaire (SAQ) (26), the Patient Safety Culture in Healthcare Organizations Survey (PSCHO) (9), and the Safety Climate Scale (27). However, again each of these questionnaires assesses a different number and combination of dimensions (ranging from one to 12), vary in length (ranging from 13 to 48 items), and have been designed for particular settings or contexts (28).

Scoring of commonly employed surveys, such as the HSOPS (25), presents further challenges as results can vary depending upon the strategy and computational method selected. While the Agency for Healthcare Research and Quality (AHRQ) recommends for HSOPS that the percentage of positive responses be computed to interpret the 12-dimensional scores, two alternative aggregation methods have been identified in the literature, leading to potential bias when comparing results between studies, hospitals and countries (29). Notably, Giai et al. (29) identified the heterogeneity of results obtained by the three scoring approaches used to assess safety culture in a French university hospital, showing that dimensional score values, as well as their corresponding rankings, varied considerably across the different scoring methods. For example, for the HSOPS dimension “teamwork within hospital units” the score for the worst performing department based on percent positive scores, increased by more than 10% using averaged individual sums (29). This study highlights that healthcare decision makers must consider comparing HSOPS results within and between organizations with great caution, and that agreement must first be reached on a consistent scoring approach.

Additionally, different versions exist for numerous safety culture surveys, including short and long versions (e.g., SAQ 36-item short form and SAQ 60-item full-form), and versions for specific contexts (e.g., HSOPS for hospitals, medical offices, ambulatory surgery centers, nursing homes, and community pharmacies). Further, both the HSOPS and SAQ have undergone major revisions in recent years. The HSOPS 2.0 was released in 2019 and involved deleting, rewording and adding multiple items (25). Furthermore, in 2019, the SAQ was superseded by the Integrated SCORE (Safety, Communication, Operational Reliability & Engagement Survey) (30), which removed one of the original dimensions and added a number of others with a greater focus on staff wellbeing, an issue to be discussed further in this paper. Brian Sexton, co-developer of the SAQ, stated that the older surveys needed to be updated as “they were not intended for use in today's healthcare environment” and had “limited evidence of reliability and validity” (31). However, the Integrated SCORE, also co-developed by Sexton, is no longer freely available, so it is unclear the extent to which this survey will be taken up by hospitals and researchers. On the other hand, transition to the HSOPS 2.0 appears to have been more positive, with countries including Australia developing their own context-specific version (the A-HSOPS 2.0) and a toolkit developed to support its implementation (32). This raises the question though of how comparable the results are between different survey versions, particularly when it comes to international benchmarking.

Further, while the use of questionnaires is practical for simply capturing data from a larger group of participants or staff, one major issue is that the exclusive reliance on quantitative data fails to capture and expose rich insights into the dimensions of culture (33). For example, questionnaires tend to only capture superficial artifacts and beliefs, rather than the underlying shared assumptions which are argued to comprise the culture of an organization (34). Consequently, some researchers argue that a more valid approach to assessing safety culture is to incorporate qualitative methods in addition to questionnaires to enable greater exploration of the identified dimensions (8, 15). However, these approaches typically require more researcher involvement and resources, such as participating in fieldwork, directing narrative interviews, or conducting observational research (12). Some questionnaires, including the HSOPS 2.0 and SCORE, recognize the need for mixed-method assessment, and also recommend the inclusion of qualitative, open ended questions at the end of the survey.

### 2.3. Inconsistency in dimensionality

Safety culture is multi-faceted, and the tools which are employed to measure the concept are typically based upon the assessment of several inter-related attributes or dimensions (16, 35). However, much like the ambiguities that manifest in the definition of safety culture (36), researchers are yet to reach a consensus on the underlying dimensions that comprise safety culture (12), thereby highlighting yet another challenge faced in the field. For example, while some narrowly define safety culture as focusing on the key dimensions of unit and organizational leadership's prioritization of safety (37); others more broadly conceptualize safety culture to include sub-dimensions such as learning, reporting, and blame orientation (21, 38, 39). Sometimes, more distant dimensions are also included, such as job satisfaction (26) and staffing (2). Furthermore, dimensions comprising safety culture are usually considered highly context dependant (40), varying by industry and even organization (41).

In an attempt to identify the fundamental dimensions of safety culture in healthcare, Flin et al. (16) reviewed 12 quantitative studies in healthcare of safety culture to identify its fundamental dimensions. The 73 safety culture dimensions identified across these 12 studies were re-categorized by the researchers into 10 distinct themes: management/supervision; safety systems; risk perception; job demands; reporting/speaking up; safety attitudes/behaviors; communication/feedback; teamwork; personal resources (such as stress); and organizational factors. In this study, management commitment to safety emerged as the most frequently measured safety culture dimension. More recently, Halligan and Zecevic (12) reviewed 113 articles which explored the dimensions of safety culture in healthcare. In this study, they found that the six most frequently cited dimensions were: leadership commitment to safety, open communication founded on trust; organizational learning; a non-punitive approach to adverse event reporting and analysis; teamwork; and a shared belief in the importance of safety. Organizational learning was identified as an important theme that was not specifically identified as a separate dimension in the Flin et al. review (16). However, for both reviews there was a lack of detail on how dimensions were identified, and in turn how they mapped to the safety culture tools they reviewed.

In a more recent systematic review assessing the dimensions of safety culture, Churruca et al. (15) assessed 694 studies (including quantitative, qualitative and mixed-methods studies) to identify the most commonly utilized approaches to assessing safety culture in healthcare, and the dimensions of safety culture captured through these processes. A comprehensive thematic analysis identified 11 dimensional themes present across studies, including: leadership; perceptions of safety; teamwork and collaboration; safety systems; prioritization of safety; resources and constraints; reporting and just culture; openness; learning and improvement; awareness of human limits; and wellbeing (15). Table 1 provides a summary of the 11 themes and the number of studies identified incorporating each theme. As shown in this table, the most commonly assessed dimensional themes present in over half of the current approaches to assessing safety culture include: leadership; perceptions of safety; teamwork and collaboration; safety systems; prioritization of safety; and resources and constraints (15).

**Table 1:** Safety culture dimensional themes.

**Theme**	**Definition**	**No. studies/surveys used in (%)**
Leadership	Leadership, their support, and commitment to safety.	85 (77.3)
Perceptions of safety	Perceptions of how safe the organization is.	65 (59.1)
Teamwork and collaboration	Working together as a team and coordination of care among staff.	61 (55.5)
Safety Systems	Systems, procedures and processes exist that facilitate patient safety (eg, rewards, reporting systems).	58 (52.7)
Prioritization of safety	Shared belief, behaviors and norms in which staff in the work area prioritize and value safety.	59 (53.6)
Resources and constraints	Resources for safety including staffing, equipment, lack of time and training.	58 (52.7)
Reporting and just culture	Willingness to report and a culture that does not assign blame.	54 (49.1)
Openness	Open communication, staff feeling comfortable to express their issues or concerns and question behaviors.	54 (49.1)
Learning and improvement	A focus on learning from mistakes, responding to, and improving systems.	51 (46.4)
Awareness of human limits	Awareness of individual ability to be safe and how that can be limited by various factors (e.g., fatigue).	24 (21.8)
Wellbeing	Job satisfaction, burnout and other psychosocial factors.	17 (15.5)

% calculated on n = 110; results adapted from Churruca et al. (15).

As shown in Table 1, staff wellbeing has been the least frequently assessed dimensional theme, present in less than a quarter of available tools (15, 42). While safety culture improvement efforts have traditionally been concentrated on interdisciplinary teamwork and patient safety education, recent research has identified that addressing staff wellbeing factors, especially health care worker burnout, may also play an important role (43–46). Burnout refers to the ongoing and unmitigated stress response that results in symptoms of depersonalization, emotional exhaustion, and a decreased sense of personal accomplishment (47). Burnout is one of the most prevalent staff wellbeing problems that healthcare professionals currently face, given the challenges imposed by the nature of clinical work, time constraints, lack of control over work processes, and the higher work demands elicited from the COVID-19 pandemic (45, 48). Recognizing that >30% of frontline healthcare staff are experiencing burnout, Sexton et al. added a greater focus on staff wellbeing to the Integrated SCORE (31). However, further work is needed to understand whether staff wellbeing should be studied as a dimension or outcome of an organization's safety culture.

### 2.4. Response rates

Another challenge when using questionnaires to assess safety culture is the need to obtain sufficient response rates. Low response rates are particularly problematic as they can increase bias, where non-responders may be systematically different from responders (49). An overall response rate above 60% is often believed to be needed in order to establish sufficient reliability and validity of the data captured (50). Some researchers argue that anything less is considered more of an assessment of “*opinion*” rather than “*culture*” (51).

Low response rates are increasingly being reported due to duplicative survey efforts, creating survey fatigue, and isolated datasets that do not produce a consistent snapshot of safety culture (50, 52, 53). Since the COVID-19 pandemic, additional time constraints, lack of resources and survey fatigue are being reported, and thus the need for survey integration has never being more acute (31).

## 3. Conclusions and recommendations

Although safety culture surveys offer practical and time-efficient tools appealing to quality improvement and international benchmarking efforts, there remains no “gold standard” for measuring safety culture, with no one survey comprehensively evaluating all the important aspects of safety culture (8). Furthermore, variations in survey versions and scoring methods limits the capacity for comparison across studies and counties, which is a factor that makes surveys appealing in the first instance.

In response to the issues we have highlighted, we first recommend using well validated surveys of safety culture followed up by qualitative methods, such as interviews or focus groups, to enrich the exploration of complex issues related to safety culture, identify priority dimensions, and provide insight into areas for improvement (14, 15). We also recommend that staff wellbeing should be regularly assessed alongside measures of safety culture and patient safety outcomes to further advance our understanding of how safety is enacted in pressured healthcare environments. The issue of survey fatigue in many hospitals, also points to the broader need to reduce duplicative survey efforts and for a more streamlined and consistent survey approach (31). Moving to an agreed gold standard survey approach across healthcare settings would certainly make benchmarking more reliable. Research has also pointed to some strategies that are available to assist in increasing response rates, such as distributing the questionnaire in person during training sessions or staff meetings, or by allocating a local champion who can motivate non-responders to consider participating (50).

While measuring patient safety culture is a key component of many OECD countries' national patient safety strategies and the topic of a large body of research (11), the next steps for improving safety culture, health system performance and outcomes for staff and patients based on its measurement are less clear. Measuring safety culture should be considered as a starting point from which improvement actions and patient safety changes emerge (2). Systematized data feedback for all who contribute to measurement is recommended, combined with problem solving, action planning and monitoring (2). Team training and team communication skills, executive walk arounds, and intervention strategies combining adaptive interventions (such as continuous learning) with technical interventions (such as clinical care algorithms) have been shown to improve patient safety and quality (54–58). Organizational strategies with bottom-up organizational and employee learning from behavioral outcomes, conducive enabling factors, and consistency over time and effective leadership are also key elements (3, 22). One promising bottom-up strategy shown to improve patient safety is safety huddles. Although huddles were originally designed to learn from errors and adverse events (known as “Safety-I”), huddles are also now being used to support learning for improvement based on situations where work goes well (Safety-II) (59), by including reflection time to allow staff to talk about and learn from things that went well. Based on the latest evidence, such safety-II-inspired huddles could also be considered to lead to improvements in safety culture (60).

Investigating issues and complexities to safety culture assessment in healthcare is a relatively young research field which needs to develop in line with the rapid changes in different healthcare systems. There are varying challenges from high to low-income countries and contexts ranging from primary care, nursing homes, homecare and specialized hospital services. We argue that a continuous critical reflection is needed in this field to keep assessment methods, instruments and approaches relevant and targeted. Keeping instruments and implementation guidance on open access and available is recommended to increase use and enable practice improvement worldwide. That is crucial of we are to encourage widespread application in poorly-resourced settings. This is also a way to support UN goal three: of sustainable development promoting good health and wellbeing for all at all ages.

## Data availability statement

The original contributions presented in the study are included in the article/supplementary material, further inquiries can be directed to the corresponding author.

## Author contributions

LE had the idea and developed the first draft of the article with EF, which was further developed in close collaboration with PH and SW. All authors contributed to the revision and have approved the final version of the article.

## References

[1] Lawati MHA Dennis S Short SD Abdulhadi NN. Patient safety and safety culture in primary health care: a systematic review. BMC Fam Pract. (2018) 19:1–12. 10.1186/s12875-018-0793-7 29960590 PMC6026504

[2] Nieva V Sorra J. Safety culture assessment: a tool for improving patient safety in healthcare organizations. BMJ Qual Saf. (2003) 12:ii17–23. 10.1136/qhc.12.suppl_2.ii17 14645891 PMC1765782

[3] Bates DW Levine DM Salmasian H Syrowatka A Shahian DM Lipsitz S. The safety of inpatient health care. New Engl J Med. (2023) 388:142–53. 10.1056/NEJMsa2206117 36630622

[4] O'Donovan R Ward M De Brún A McAuliffe E. Safety culture in health care teams: a narrative review of the literature. J Nurs Manag. (2019) 27:871–83. 10.1111/jonm.12740 30556612

[5] International Nuclear Safety Advisory Group (INSAG). Basic safety principles for nuclear power plant. Vienna: International Atomic Energy Agency. (1988).

[6] Kohn LT. To err is human: building a safer health system. Committee on Quality of Health Care in America, Institute of Medicine. (1999).

[7] Ginsburg LR Tregunno D Norton PG Mitchell JI Howley H. ‘Not another safety culture survey': using the Canadian patient safety climate survey (Can-PSCS) to measure provider perceptions of PSC across health settings. BMJ Qual Saf. (2013) 2013:bmjqs-2013-002220. 10.1136/bmjqs-2013-002220 24122954 PMC3913119

[8] Hogden A, Ellis, LA, Churruca, K, Bierbaum, M,. Safety Culture Assessment in Health Care: A review of the literature on safety culture assessment modes. ACSQHC. (2017). Available online at: https://www.safetyandquality.gov au/wpcontent/uploads/2017/10/Safety-Culture-Assessment-in-Health-Care-A-review-of-the-literature-on-safety-culture-assessment-modes. pdf

[9] Singer S Meterko M Baker L Gaba D Falwell A Rosen A. Workforce perceptions of hospital safety culture: development and validation of the patient safety climate in healthcare organizations survey. Health Serv Res. (2007) 42:1999–2021. 10.1111/j.1475-6773.2007.00706.x 17850530 PMC2254575

[10] Barnes SA Compton J Saldaña M Tecson KM Hastings C Kennerly DA. Development and Testing of Baylor Scott & White Health's “Attitudes and Practices of Patient Safety Survey”. Baylor University Medical Center Proceedings (2016). Taylor & Francis. 10.1080/08998280.2016.11929472 PMC5023285 27695163

[11] de Bienassis K Klazinga NS. Developing international benchmarks of patient safety culture in hospital care. Organisation for Economic Co-operation and Development. (2022). Contract No.: 134.

[12] Halligan M Zecevic A. Safety culture in healthcare: a review of concepts, dimensions, measures and progress. BMJ Qual Saf. (2011) 20:338–43. 10.1136/bmjqs.2010.040964 21303770

[13] Reason J. Managing the Risks of Organizational Accidents. London: Routledge (2016).

[14] Cooper MD. The safety culture construct: theory and practice. safety cultures, safety models: taking stock and moving forward. Jama. (2018) 2018:47–61. 10.1007/978-3-319-95129-4_5

[15] Churruca K Ellis LA Pomare C Hogden A Bierbaum M Long JC. Dimensions of safety culture: a systematic review of quantitative, qualitative and mixed methods for assessing safety culture in hospitals. Bmj Open. (2021) 11:7. 10.1136/bmjopen-2020-043982 34315788 PMC8317080

[16] Flin R Burns C Mearns K Yule S Robertson E. Measuring safety climate in health care. BMJ Qual Saf. (2006) 15:109–15. 10.1136/qshc.2005.014761 16585110 PMC2464831

[17] Kalteh HO Mortazavi SB Mohammadi E Salesi M. The relationship between safety culture and safety climate and safety performance: a systematic review. Int J Occupat Safety Ergonomics. (2021) 27:206–16. 10.1080/10803548.2018.1556976 30526393

[18] Arzahan ISN Ismail Z Yasin SM. Safety culture, safety climate, and safety performance in healthcare facilities: a systematic review. Saf Sci. (2022) 147:105624. 10.1016/j.ssci.2021.10562427490260

[19] Morello RT Lowthian JA Barker AL McGinnes R Dunt D Brand C. Strategies for improving patient safety culture in hospitals: a systematic review. BMJ Qual Saf. (2013) 22:11–8. 10.1136/bmjqs-2011-000582 22849965

[20] Anderson M. Contemporary Ergonomics and Human Factors. London: Taylor & Francis (2013).

[21] Cooper MD. Towards a model of safety culture. Saf Sci. (2000) 36:111–36. 10.1016/S0925-7535(00)00035-7

[22] Bisbey TM Kilcullen MP Thomas EJ Ottosen MJ Tsao K Salas E. Safety culture: An integration of existing models and a framework for understanding its development. Hum Factors. (2021) 63:88–110. 10.1177/0018720819868878 31424954

[23] Hogden A Churruca K Bierbaum M. Safety culture assessment in health care: a review of the literature on safety culture assessment modes (2017).

[24] Vu T De Cieri H. A review and evaluation of safety culture and safety climate measurement tools. WorkSafe Victoria: Monash University (2015). p. 68.29747612

[25] Agency for Healthcare Research Quality. Surveys on Patient Safety Culture (SOPS) Hospital Survey. Rockville, MD: Agency for Healthcare Research and Quality (2021). Available online at: https://www.ahrq.gov/sops/surveys/hospital/index.html (accessed April 9, 2023).

[26] Sexton JB Helmreich RL Neilands TB Rowan K Vella K Boyden J. The Safety Attitudes Questionnaire: psychometric properties, benchmarking data, and emerging research. BMC Health Services Res. (2006) 6:44. 10.1186/1472-6963-6-44 16584553 PMC1481614

[27] Thomas EJ Sexton JB Neilands TB Frankel A Helmreich RL. The effect of executive walk rounds on nurse safety climate attitudes: a randomized trial of clinical units[ISRCTN85147255] [corrected]. BMC Health Serv Res. (2005) 5:28. 10.1186/1472-6963-5-28 15823204 PMC1097728

[28] Agency for Healthcare Research Quality. Medical Office Survey on Patient Safety Culture. (2013). Available online at: https://www.ahrq.gov/sops/surveys/medical-office/index.html (accessed February 9, 2023).

[29] Giai J Boussat B Occelli P Gandon G Seigneurin A Michel P. Hospital survey on patient safety culture (HSOPS): variability of scoring strategies. Int J Qual Health Care. (2017) 29:685–92. 10.1093/intqhc/mzx086 28992144

[30] Sexton B Franke A Leonard L Adair K. SCORE: Assessment of your work setting safety, communication, operational reliability, and engagement. Technical Report (2017).33993868

[31] Healthcare SR. The Integrated SCORE (Safety, Communication, Operational Reliability & Engagement Survey) Survey for Organizational Learning and Improvement. Evergreen, CO: Safe & Reliable Healthcare (2014).

[32] Australian Commission on Safety and Quality in Health Care. Measuring Patient Safety Culture: Technical specifications for the Australian HSOPS 2.0 (2021).

[33] Schein EH. Organizational Culture and Leadership. London: John Wiley & Sons (2010).

[34] Schein EH. “Commentary: Sense and nonsense about culture and climate,” In: *Handbook of Organizational Culture and Climate.* Thousand Oaks, CA: Sage (2000).

[35] Singla AK Kitch BT Weissman JS Campbell EG. Assessing patient safety culture: a review and synthesis of the measurement tools. J Pat Safety. (2006) 2006:105–15. 10.1097/01.jps.0000235388.39149.5a27820412

[36] Reader TW Noort MC Shorrock S Kirwan B. Safety sans frontières: an international safety culture model. Risk Analy. (2015) 35:770–89. 10.1111/risa.12327 25683474

[37] Zohar D A. group-level model of safety climate: testing the effect of group climate on microaccidents in manufacturing jobs. J Appl Psychol. (2000) 85:587. 10.1037/0021-9010.85.4.587 10948803

[38] Reason J. Managing the Risks of Organizational Accidents. Aldershot, UK: Ashgate Publishing Company (1997).

[39] Hofmann DA Mark B. An investigation of the relationship between safety climate and medication errors as well as other nurse and patient outcomes. Pers Psychol. (2006) 59:847–69. 10.1111/j.1744-6570.2006.00056.x

[40] Ginsburg L Gilin D Tregunno D Norton PG Flemons W Fleming M. Advancing measurement of patient safety culture. Health Serv Res. (2009) 44:205–24. 10.1111/j.1475-6773.2008.00908.x 18823446 PMC2669635

[41] Coyle IR Sleeman SD Adams N. Safety climate. J Safety Res. (1995) 26:247–54. 10.1016/0022-4375(95)00020-Q

[42] Montgomery AJ Van der Doef M Panagopoulou E Leiter MP. Connecting health care worker wellbeing, patient safety and organizational change: the triple challenge. connecting healthcare worker wellbeing, patient safety and organisational change. The Triple Chall. (2020) 2020:1–7. 10.1007/978-3-030-60998-6_1

[43] Campione J Famolaro T. Promising practices for improving hospital patient safety culture. Jt Comm J Qual Patient Saf. (2018) 44:23–32. 10.1016/j.jcjq.2017.09.001 29290243

[44] Lu L Ko YM Chen HY Chueh JW Chen PY Cooper CL. Patient safety and staff wellbeing: organizational culture as a resource. Int J Environ Res Public Health. (2022). 19:6. 10.3390/ijerph19063722 35329410 PMC8953540

[45] Hall LH Johnson J Watt I Tsipa A O'Connor DB. Healthcare staff wellbeing, burnout, and patient safety: a systematic review. PLoS One. (2016) 11:e0159015. 10.1371/journal.pone.0159015 27391946 PMC4938539

[46] Garcia CdL Abreu LCd Ramos JLS Castro CFDd Smiderle FRN Santos JAd. Influence of burnout on patient safety: systematic review and meta-analysis. Medicina. (2019) 55:553. 10.3390/medicina55090553 31480365 PMC6780563

[47] Salyers MP Bonfils KA Luther L Firmin RL White DA Adams EL. The relationship between professional burnout and quality and safety in healthcare: a meta-analysis. J Gen Intern Med. (2017) 32:475–82. 10.1007/s11606-016-3886-9 27785668 PMC5377877

[48] Bridgeman PJ Bridgeman MB Barone J. Burnout syndrome among healthcare professionals. Bull Am Soc Hospital Pharmacists. (2018) 75:147–52. 10.2146/ajhp170460 29183877

[49] Schalm RL Kelloway EK. The relationship between response rate and effect size in occupational health psychology research. J Occup Health Psychol. (2001) 6:160. 10.1037/1076-8998.6.2.160 11326727

[50] Ellis LA Pomare C Churruca K Carrigan A Meulenbroeks I Saba M. Predictors of response rates of safety culture questionnaires in healthcare: a systematic review and analysis. BMJ Open. (2022) 12:e065320. 10.1136/bmjopen-2022-065320 36113948 PMC9486325

[51] Pronovost P Sexton B. Assessing Safety Culture: Guidelines and Recommendations. London: BMJ Publishing Group Ltd (2005). p. 231-3. 10.1136/qshc.2005.015180 PMC1744052 16076784

[52] Cunningham CT Quan H Hemmelgarn B Noseworthy T Beck CA Dixon E. Exploring physician specialist response rates to web-based surveys. BMC Med Res Methodol. (2015) 15:1–8. 10.1186/s12874-015-0016-z 25888346 PMC4404667

[53] Asch DA Jedrziewski MK Christakis NA. Response rates to mail surveys published in medical journals. J Clin Epidemiol. (1997) 50:1129–36. 10.1016/S0895-4356(97)00126-1 9368521

[54] Weaver SJ Lubomksi LH Wilson RF Pfoh ER Martinez KA Dy SM. Promoting a culture of safety as a patient safety strategy: a systematic review. Ann Int Med. (2013) 158:369–74. 10.7326/0003-4819-158-5-201303051-00002 23460092 PMC4710092

[55] Sexton JB Sharek PJ Thomas EJ Gould JB Nisbet CC Amspoker AB. Exposure to Leadership WalkRounds in neonatal intensive care units is associated with a better patient safety culture and less caregiver burnout. BMJ Qual Saf. (2014) 23:814–22. 10.1136/bmjqs-2013-002042 24825895 PMC4167964

[56] Sexton JB Adair KC Leonard MW Frankel TC Proulx J Watson SR. Providing feedback following Leadership WalkRounds is associated with better patient safety culture, higher employee engagement and lower burnout. BMJ Qual Saf. (2018) 27:261–70. 10.1136/bmjqs-2016-006399 28993441 PMC5867443

[57] Leroy H Dierynck B Anseel F Simons T Halbesleben JR McCaughey D. Behavioral integrity for safety, priority of safety, psychological safety, and patient safety: A team-level study. J Appl Psychol. (2012) 97:1273. 10.1037/a0030076 22985115

[58] Ferorelli D Benevento M Vimercati L Spagnolo L De Maria L Caputi A. Improving healthcare workers' adherence to surgical safety checklist: the impact of a short training. Front Public Health. (2022) 9:2430. 10.3389/fpubh.2021.732707 35211450 PMC8860967

[59] Braithwaite J Wears RL Hollnagel E. Resilient health care: turning patient safety on its head. Int J Qual Health Care. (2015) 27:418–20. 10.1093/intqhc/mzv063 26294709

[60] Wahl K Stenmarker M Ros A. Experience of learning from everyday work in daily safety huddles-a multi-method study. BMC Health Serv Res. (2022) 22:1101. 10.1186/s12913-022-08462-9 36042516 PMC9424837

